# Ferroptosis-related long non-coding RNA signature predicts the prognosis of hepatocellular carcinoma

**DOI:** 10.18632/aging.204073

**Published:** 2022-05-12

**Authors:** Xin Yang, Minhui Mei, Jingze Yang, Jinlu Guo, Fan Du, Shi Liu

**Affiliations:** 1 Department of Gastroenterology, Union Hospital, Tongji Medical College, Huazhong University of Science and Technology, Wuhan, China

**Keywords:** hepatocellular carcinoma, ferroptosis, long non-coding RNAs, immune infiltration, prognosis

## Abstract

Background: Hepatocellular Carcinoma (HCC) is a highly heterogeneous malignant tumor, and its prognostic prediction is extremely challenging. Ferroptosis is a cell mechanism dependent on iron, which is very significant for HCC development. Long non-coding RNA (lncRNA) is also linked to HCC progression. This work aimed to establish a prognosis risk model for HCC and to discover a possible biomarker and therapeutic target.

Methods: The Cancer Genome Atlas (TCGA) database was used to obtain RNA-seq transcriptome data and clinic information of HCC patients. Firstly, univariate Cox was utilized to identify 66 prognostic ferroptosis-related lncRNAs. Then, the identified lncRNAs were further included in the multivariate Cox analysis to construct the prognostic model. Eventually, we performed quantitative polymerase chain reaction (q-PCR) to validate the risk model.

Results: We established a prognostic seventeen-ferroptosis-related lncRNA signature model. The signature could categorize patients into two risk subgroups, with the low-risk subgroup associated with a better prognosis. Additionally, the area under the curve (AUC) of the lncRNAs signature was 0.801, indicating their reliability in forecasting HCC prognosis. Risk score was an independent prognostic factor by regression analyses. Gene set enrichment analysis (GSEA) analyses demonstrated a remarkable enrichment of cancer-related and immune-related pathways in the high-risk group. Besides, the immune status was decreased in the high-risk group. Eventually, three prognostic lncRNAs were validated in human HCCLM3 cell lines.

Conclusions: The risk model based on seventeen-ferroptosis-related lncRNA has significant prognostic value for HCC and may be therapeutic targets associated with ferroptosis in clinical ways.

## INTRODUCTION

The occurrence of liver cancer ranks sixth among malignant tumors and fourth among the causes of tumor-associated death in the world [1]. Hepatocellular carcinoma (HCC) is the most familiar form of primary hepatic carcinoma and is related to some common causes, including chronic hepatitis, alcoholism, NAFLD, and exposure to food toxins like aflatoxins [2]. HCC has low survival rates and is a highly heterogeneous disease; the survival rate at 5 years is only 18% in the United States [2–4]. The complex etiology and the high heterogeneity of HCC cause a challenge to the prediction of prognosis. Thus, considering the limitations of current treatment strategies for HCC, it is necessary to develop a new prognostic model.

In the past few decades, research on tumor ferroptosis has increased rapidly. Unlike apoptosis and autophagy, ferroptosis is iron-dependent and regulates cell death through the lethal accumulation of lipid peroxidation [5–7]. Abnormal iron metabolism is a risk factor for cancer and will facilitate tumor growth. Relative to normal cells, cancer cells are addicted to iron, and they are excessively dependent on iron to promote proliferation [8]. In the past few years, inducing ferroptosis has become a potentially beneficial treatment that can make cancer cells die, especially for those malignant tumors that resist traditional therapy [9, 10].

Long non-coding RNAs (lncRNAs), lacking protein-coding ability and spanning over 200 nucleotides in length, can regulate gene expression [11, 12]. In addition to gene regulation, lncRNA is also involved in a variety of biological regulatory processes, like those associated with tumor occurrence, tumor development, and tumor metastasis [13]. Currently, molecular risk signatures, particularly lncRNA signatures, have been studied as prognostic indicators of cancer development [14]. There are, however, few studies based on sequences that assess ferroptosis-associated lncRNA signatures and their relationship with overall survival (OS) in HCC patients.

In this work, we established a molecular signature model with seventeen prognostic ferroptosis-related lncRNAs based on the Cancer Genome Atlas (TCGA) data. Then, we assessed the model's ability to predict HCC prognosis and investigated the relationship between clinical characteristics and the seventeen prognostic ferroptosis-related lncRNAs. Moreover, gene set enrichment analysis (GSEA) and association analyses with immune cell infiltration were utilized to explore the immune-associated characteristics of this molecular signature model. Finally, three prognostic lncRNAs (LINC00942, ZFPM2-AS1, and LINC00205) were validated in human HCCLM3 cell lines.

## METHODS

### Data availability

In this study, 376 patients were recruited to acquire their RNA-sequence data, which was extracted from TCGA-LIHC databases on June 22, 2021 (https://portal.gdc.cancer.gov/repository). Table 1 displays the clinical characteristics of the patients. The corresponding ferroptosis-associated genes were available from FerrDb [15], which is a consortium based on the web, providing the latest and all-round information on ferroptosis markers, their regulatory molecules, as well as associated diseases. In all, 246 ferroptosis-associated genes (Supplementary Table 1) were identified. We used the “limma” software package to calculate the correlation of expression between lncRNAs and ferroptosis-associated genes and identify ferroptosis-related lncRNAs (|Pearson R| > 0.4 and *p* <0.001). Clinical and pathological data of HCC patients, including gender, age, grade, stage, time, status, and TMN were collected. The remarkable differential expression of ferroptosis-related lncRNAs was defined as |log2FC|≥1 and false discovery rate (FDR) <0.05. Firstly, the function of up-regulated and down-regulated ferroptosis-associated differentially expressed genes (DEGs) were explored. Then, Gene ontology (GO) was used to assess the biological pathways related to the DEGs. Based on the data from Kyoto Encyclopedia of Genes and Genomes (KEGG), the R software and ggplot2 were used to analyze the biological processes, molecular functions and cellular components controlled by differentially expressed long non-coding RNAs associated with ferroptosis.

**Table 1:** The clinical characteristics of patients in the TCGA dataset.

**Variable**	**Number of samples**
Gender	
Male/Female	254/122
Age at diagnosis	
≤65/>65/NA	224/152
Grade	
G1/G2/G3/G4/NA	55/180/123/13/5
Stage	
I/II/III/IV/NA	175/86/86/5/24
T	
T0/T1/T2/T3/T4/NA	0/185/94/81/13/3
M	
M0/M1/NA	272/4/100
N	
N0/N1/N2/N3/NA	257/4/0/0/115

### Establishment of a ferroptosis-related lncRNAs prognostic signature

The ferroptosis-related lncRNA signature was built using univariate Cox regression and multivariate Cox regression analysis. The risk scores were calculated using the following equations: $\text{Risk}\;\text{score}=\sum_{i=1}^{n}\text{Coef}i\times \text{Exp}i,$ where Coef was the coefficient of lncRNA correlated with survival and Exp was the expression level of every retained lncRNA. Based on the median cut-off value, subgroups including high-risk and low-risk groups were established from TCGA patients with HCC. We performed Kaplan-Meier survival analysis using the R packages “survMiner” and “surviva” to analyze differences in OS between the low- and high-risk subgroups based on the ferroptosis-associated lncRNA signature. The area under the curve (AUC) of time-dependent receiver operating characteristic (ROC) curves and decision curve analysis (DCA) [16] were used to assess whether the derived prognostic signs of HCC are more sensible and specific than other clinicopathological indicators. In terms of gene expressions in the prognostic model, the “limma” and “scatterplot3d” R packages were used to conduct principal component analysis (PCA) for the two risk subgroups. Univariate and multivariate Cox regression analyses were performed to evaluate whether the risk score was an independent prognostic predictor of OS when other clinical factors (age, gender, grade, stage, T stage, M stage, and N stage) were taken into account in patients with HCC. The Cytoscape software (version 3.8.2) was used to examine the link between the identified lncRNAs and ferroptosis-associated genes. Besides, a heatmap graph was used to assess the correlation between clinicopathological features and ferroptosis-related lncRNAs.

### Creating and validating a predictive nomogram

Using the R package of “rms”, the clinical features (age, gender, grade, T stage, M stage, and N stage) and risk score were utilized to construct a prognostic nomogram to predict the 1-, 3-, and 5-year OS of patients with HCC. Each variable in the nomogram scoring system was matched with a score, and the overall score was calculated by summing the scores from all variables in each sample [17]. The nomogram calibration plots were utilized to show the predictive value between the forecasted 1-, 3-, and 5-year OS and the practically observed results.

### Immunity assessment and gene expression

Based on the obtained ferroptosis-related lncRNA signature, gene set enrichment analysis (GSEA) (version 4.1.0) was used to examine the KEGG pathway analysis between the high-risk subgroup and the low-risk subgroup. In addition, based on our signature, the TIMER [18], CIBERSORT [19], CIBERSORTABS [19], QUANTISEQ [20], MCPCOUNTER [21], XCELL [22], and EPIC [23] algorithms were compared to evaluate the fraction of tumor-infiltrating immune cells in the high-risk subgroup and low-risk subgroup. A heatmap was used to find the distinction in immune response based on distinct algorithms. Additionally, previous literature was used to find a possible immunity check-point.

### Cell culture and quantitative polymerase chain reaction (q-PCR)

The normal human hepatic epithelial cell line LO2 and the HCC cell lines HCCLM3 were cultured in DMEM medium supplemented with 10% fetal bovine serum at 37°C in a humidified atmosphere with 5% CO_2_. RIZOL (Invitrogen) reagent was used to extract total RNA from cells. The cDNA was then obtained through reverse transcription with the cDNA Synthesis Mix and analyzed utilizing quantitative PCR. GAPDH was employed as an internal reference. Gene expression levels were calculated utilizing 2^−ΔΔCt^ statistic. Supplementary Table 2 displays the primer sequences used in this study.

### Statistical analysis

The PERL programming language (version 5.32.1) was used to preprocess the RNA-seq transcriptome data. R software’s Bioconductor packages (version 4.0.5) and GraphPad Prism software (Version 8.0) were also used to analyze the data. The Chi-square examination was conducted to compare the categorical variables between groups divided into low-risk and high-risk categories. In this study, the statistical significance level for each analysis was set at *P* < 0.05.

## RESULTS

### Enrichment analysis of genes associated with ferroptosis

**Figure 1:**
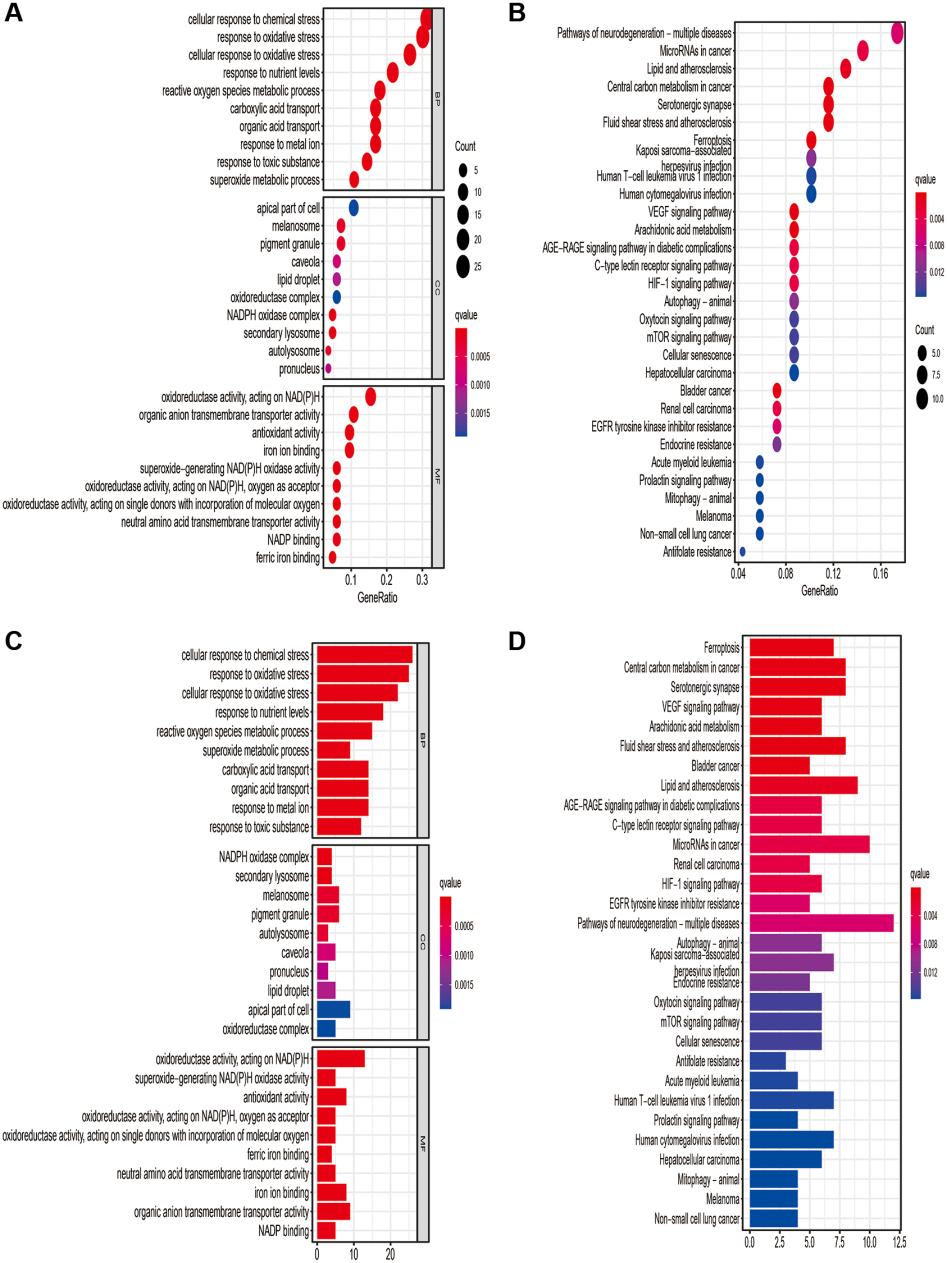
**Results of Gene Ontology (GO) and Kyoto Encyclopedia of Genes and Genomes (KEGG) analyses.** GO (**A**, **B**) and KEGG (**C**, **D**) analysis based on the ferroptosis-related differentially expressed genes.

84 DEGs (13 downregulated and 71 upregulated; Supplementary Table 3) associated with ferroptosis were found. An analysis based on KEGG found that the over-expressed genes were mainly concerned with ferroptosis, cancer-related microRNAs expressing, central carbon metabolism in cancer cells, mTOR signaling pathway, hypoxia-inducible factor (HIF)-1 signaling pathway, and the VEGF signaling pathway (Figure 1; Supplementary Table 4).

### Long non-coding RNAs prognostic signature based on ferroptosis

781 ferroptosis-associated lncRNAs were discovered (Supplementary Table 5). In the univariate COX analysis, 66 remarkable ferroptosis-associated lncRNAs were found, which were included in the multivariate COX analysis (Supplementary Table 6). Eventually, a seventeen-ferroptosis-associated lncRNA signature for predicting the prognosis of patients with HCC was constructed, including five low-risk genes (AC099850.1, LINC00205, AC026401.3, AC145207.8, and SNHG21) and twelve high-risk genes (POLH-AS1, SNHG10, AL139384.1, AL928654.1, AL603839.3, MKLN1-AS, AC090772.3, ZFPM2-AS1, AP001469.3, AC012073.1, AL031985.3, and LINC00942) (Supplementary Table 6). Then, we separated HCC patients into low- and high-risk subgroups based on the median value of the risk score.

### Validation of the prognostic value of the ferroptosis-related lncRNA signature

**Figure 2:**
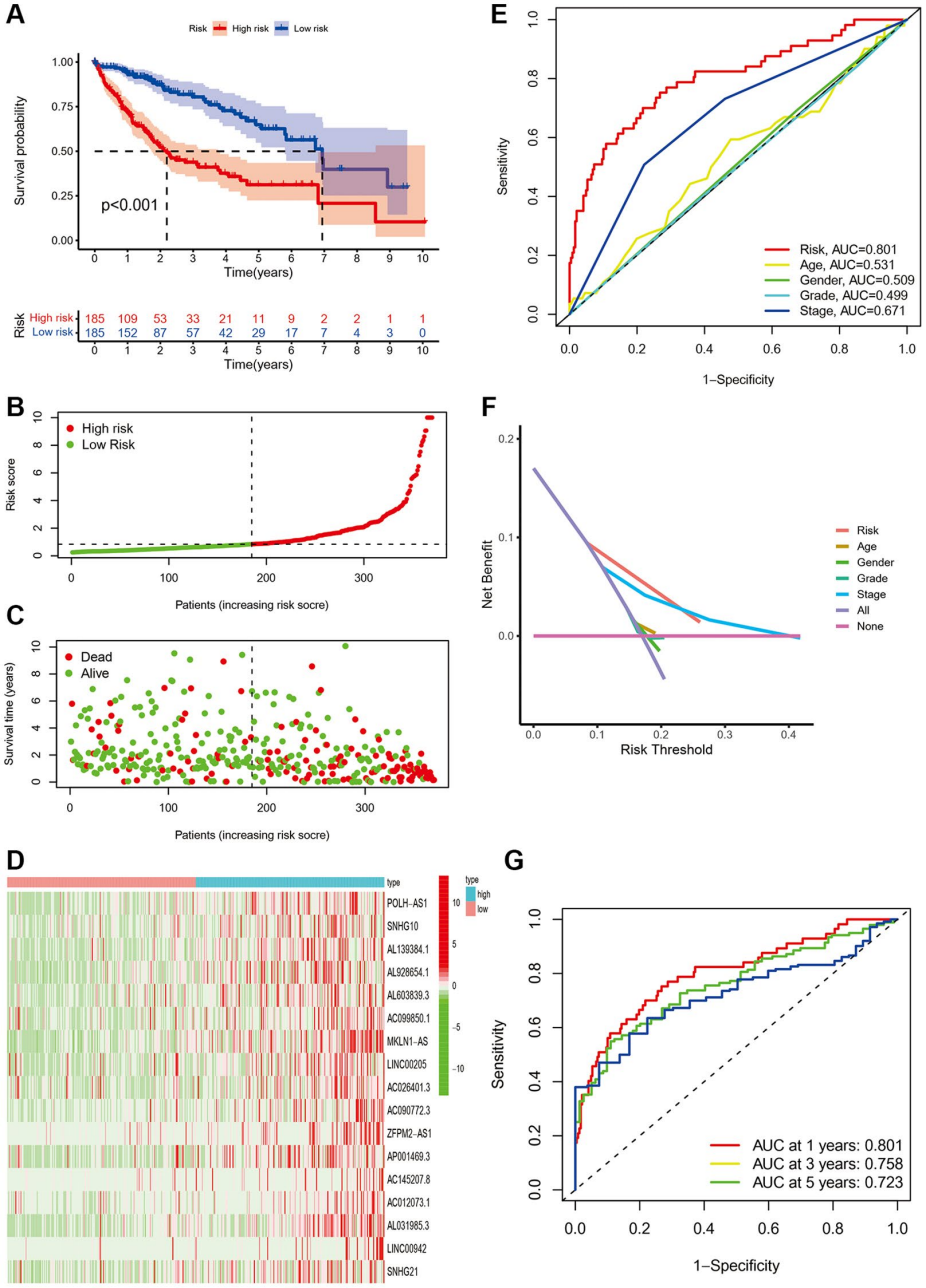
**The prognostic performance of the seventeen ferroptosis-related lncRNA signature based on TCGA cohort.** (**A**) The Kaplan-Meier analysis of overall survival in low- and high-risk groups. (**B**–**D**). The distribution of risk scores, survival status, and expression of the seventeen ferroptosis-related lncRNA risk genes. (**E**) Area under time-dependent ROC curve (AUC) of time-dependent Receiver operating characteristic curve (ROC) curves compared the prognostic accuracy of the risk score and other clinical features. (**F**) Decision curve analysis (DCA) compared the prognostic accuracy of the risk score and other clinicopathological. (**G**) AUC of time-dependent ROC curves validated the prognostic accuracy of the risk score in TCGA cohort.

The KM curve showed that patients in the low-risk group had significantly better OS than those in the high-risk group (Figure 2A, *P* < 0.001). Figure 2B demonstrated the variation of risk scores between the low-risk and high-risk groups. Figure 2C shows there were more fatalities and fewer years of survival in the high-risk group. As shown in Figure 2D, the RNA expression of the seventeen ferroptosis-associated lncRNAs was lower in the low-risk group than in the high-risk group.

**Figure 3:**
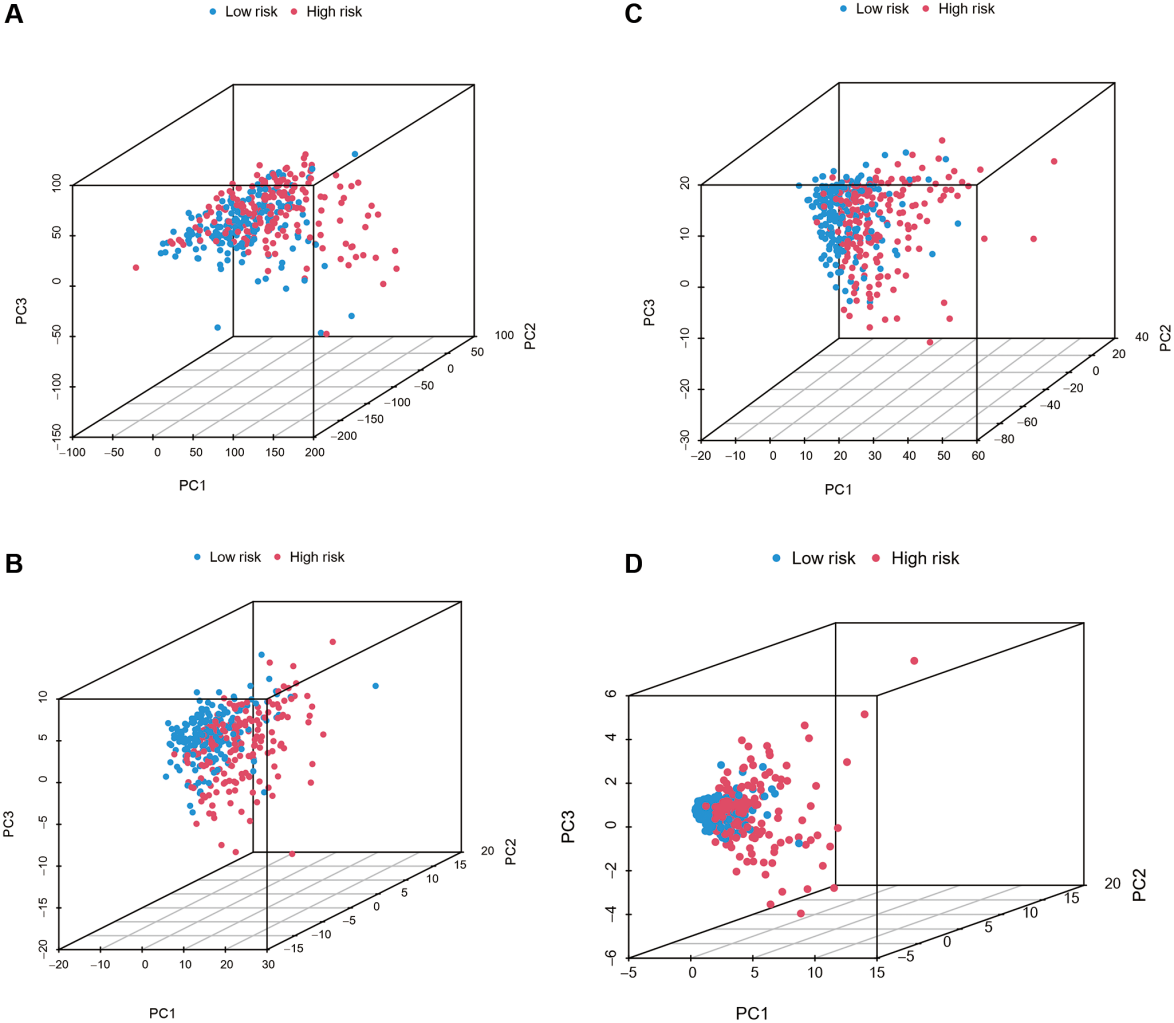
Principal component analysis between the low-risk and high-risk subgroups based on all genes, 246 ferroptosis genes, 1,271 ferroptosis-related lncRNAs, and seventeen risk genes (**A**–**D**).

Then, PCA was used to compare the low-risk and high-risk groups based on all genes, 246 ferroptosis genes, 1,271 ferroptosis-related lncRNAs, and 17 risk genes. As shown in Figure 3A–3C, the distributions of the low- and high-risk groups were relatively dispersed. However, the finding of seventeen risk genes revealed that the low- and high-risk groups had distinct distributions (Figure 3D). These findings indicate that the low-risk and high-risk group had different distributions based on the risk model.

Meanwhile, the area under the curve (AUC) of signature lncRNAs was 0.801, which is better than traditional clinicopathological characteristics in the prediction of prognosis of HCC (Figure 2E, 2F). ROC curves were used to assess the predictive power of the prognostic model, and the AUC was 0.801 at one year, 0.758 at three years, and 0.723 at five years, respectively (Figure 2G).

**Figure 4:**
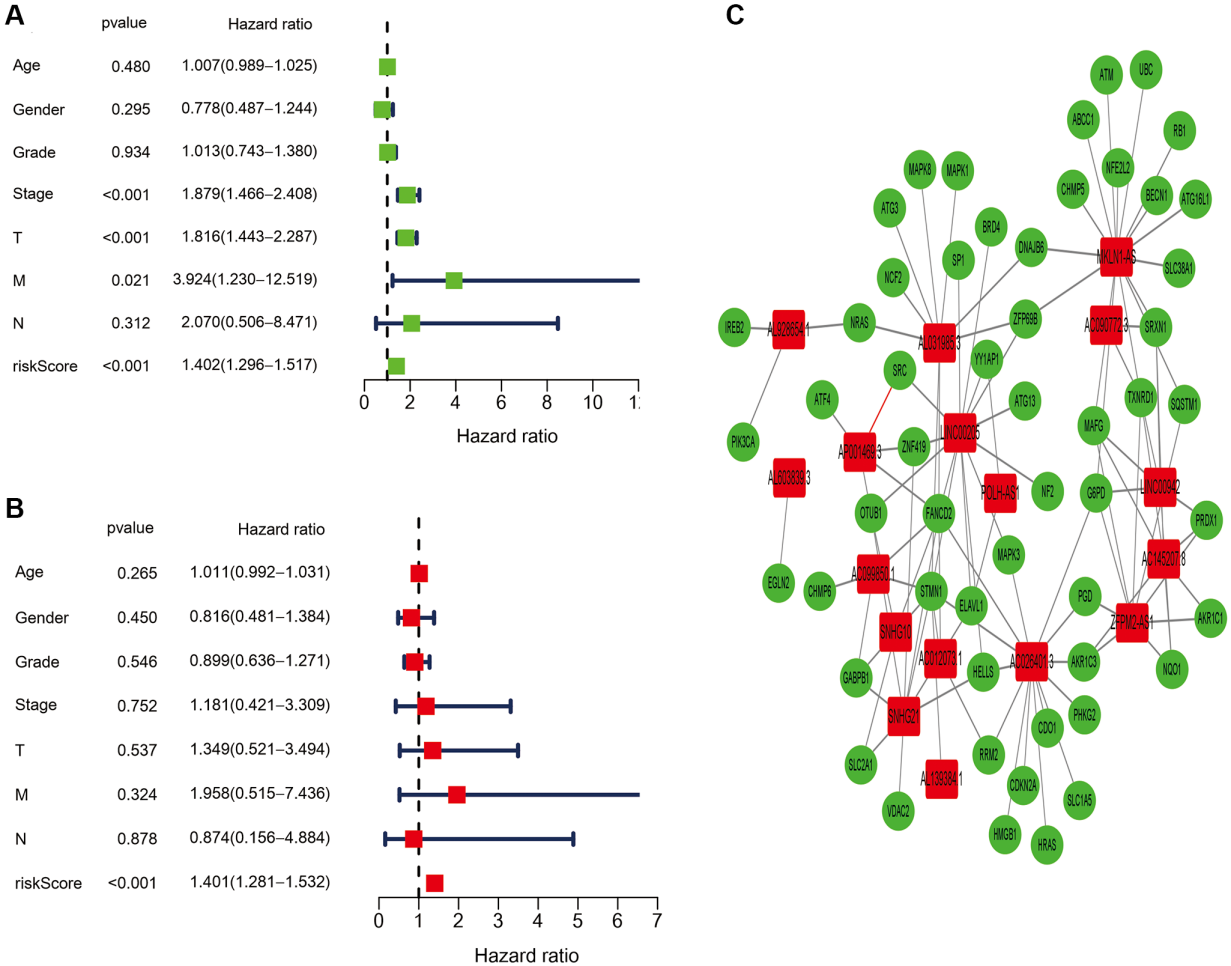
**Independent prognostic value of the ferroptosis-related lncRNAs signature.** (**A**) The results of the univariate Cox regression analysis in terms of overall survival (OS). (**B**) The results of the multivariate Cox regression analysis in terms of overall survival (OS). (**C**) The link between the identified seventeen ferroptosis-related lncRNAs and ferroptosis-related genes.

**Figure 5:**
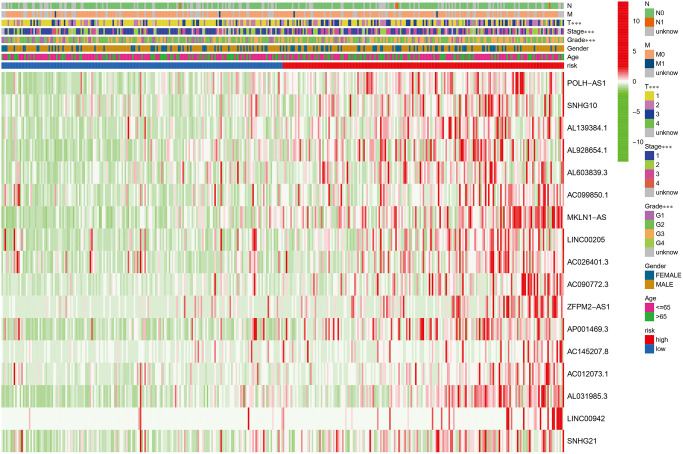
Heatmap showing the associations between clinicopathologic characteristics and risk groups, which discovered that the grade, T stage, and stage were all distributed differently between the high- and low-risk groups (green: low expression; red: high expression; ^***^P < 0.001).

Next, the risk model of 17 ferroptosis-related lncRNAs was evaluated further utilizing univariate and multivariate Cox regression analysis to see whether it may serve as an independent prognostic factor in patients with HCC. In univariate Cox regression analyses, stage, T, M and risk score were markedly associated with OS (HR = 1.879, 95% CI = 1.466−2.408, *P* < 0.001; HR = 1.816, 95% CI = 1.443−2.287, *P* < 0.001; HR = 3.924, 95% CI = 1.230−12.519, *P* = 0.021; HR = 1.402, 95% CI = 1.296−1.517, *P* < 0.001, respectively, Figure 4A). After controlling for additional confounding variables, the results of the multivariate Cox regression analysis revealed that the risk score is still an independent predictor of OS in HCC patients (HR = 1.401, 95% CI = 1.281−1.532, *P* < 0.001, Figure 4B), indicating that the predicted effect of the risk model of the ferroptosis-related lncRNAs was better than the clinicopathological parameters. Figure 4C depicts the link between identified lncRNAs and the ferroptosis-related genes. Furthermore, we created a heatmap to show the relationship between clinicopathological features and the seventeen-ferroptosis-associated lncRNA prognostic signature, and discovered that the grade, T and stage were all distributed differently between the high- and low-risk groups (Figure 5, *P* < 0.001).

**Figure 6:**
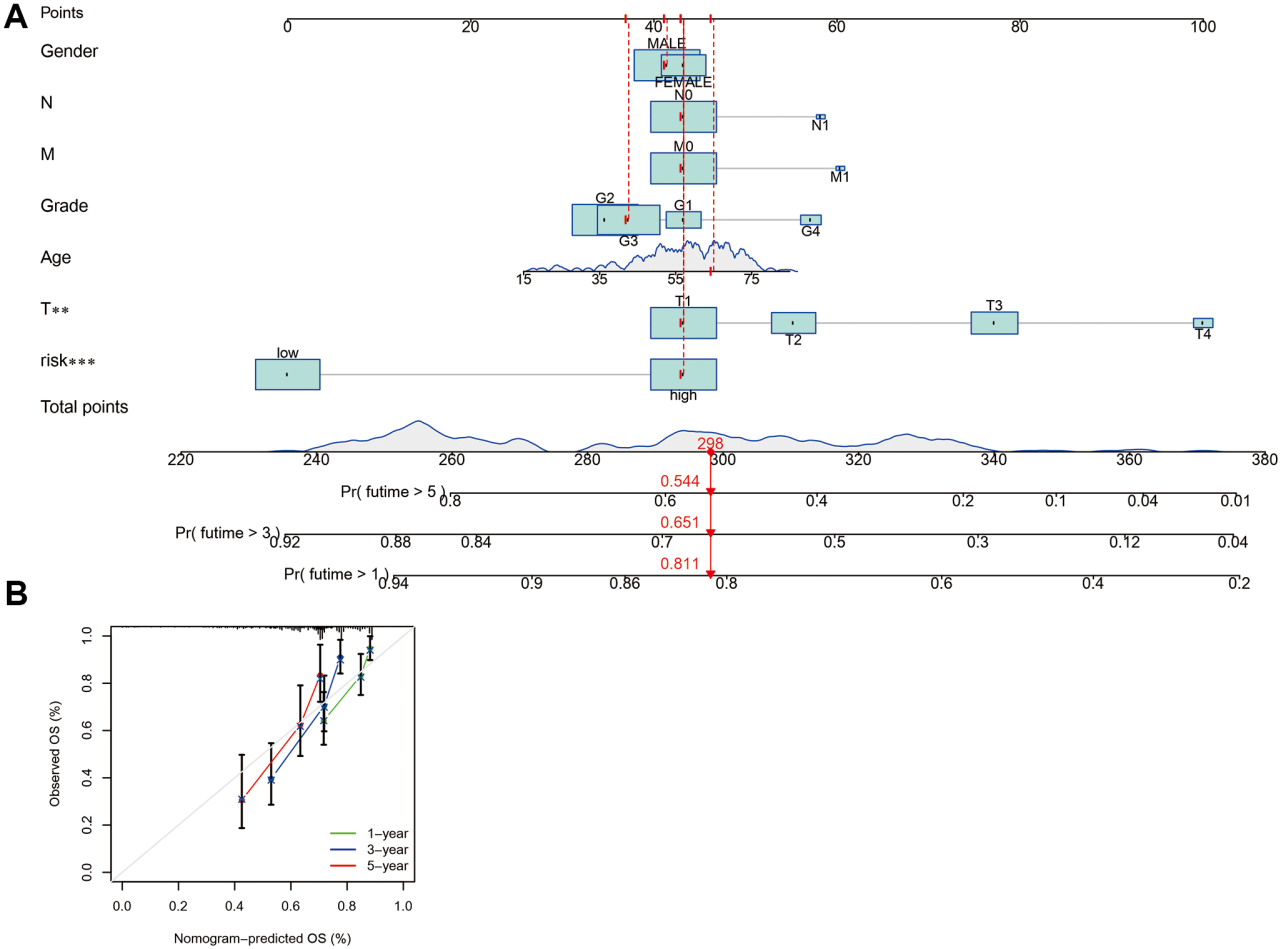
**The development and assessment of a predictive nomogram.** (**A**) The nomogram forecasts the possibility of 1-, 3-, and 5-year overall survival. (**B**) The calibration plot of the nomogram forecasts the likelihood of the 1-, 3-, and 5-year overall survival.

Given the inconvenience of the clinical value of the risk score in predicting OS rates in HCC patients, a nomogram was constructed by combining the risk score with clinicopathological features including age, gender, grade, and TMN stage to provide a reliable and quantifiable way for forecasting 1-, 3-, and 5-year survival of HCC patients (Figure 6A). The subsequent correlation plots demonstrated that the observed versus predicted rates of the 1-, 3-, and 5-year OS had excellent consistency (Figure 6B).

### Gene set enrichment analyses

**Figure 7:**
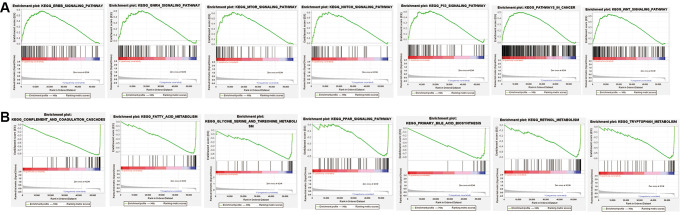
**Gene set enrichment analysis (GSEA) of low-risk subgroup and high-risk subgroup based on the ferroptosis-related lncRNAs prognostic signature.** (**A**) GSEA results demonstrate an obvious enrichment of immunoregulatory pathways against cancer in high-risk HCC patients. (**B**) GSEA results demonstrate a remarkable enrichment of amino acid metabolism in low-risk HCC patients.

GSEA results demonstrate an obvious enrichment of immunoregulatory pathways against cancer in high-risk HCC patients, such as the mTOR signaling pathway, WNT signaling pathway, ERBB signaling pathway, GNRH signaling pathway, NOTCH signaling pathway, and P53 signaling pathway. Meanwhile, GSEA results demonstrate a remarkable enrichment of amino acid metabolism in low-risk HCC patients, including glycine, serine, and threonine metabolism, as shown in Figure 7 and Supplementary Table 7.

### Expression of immunity and gene

**Figure 8:**
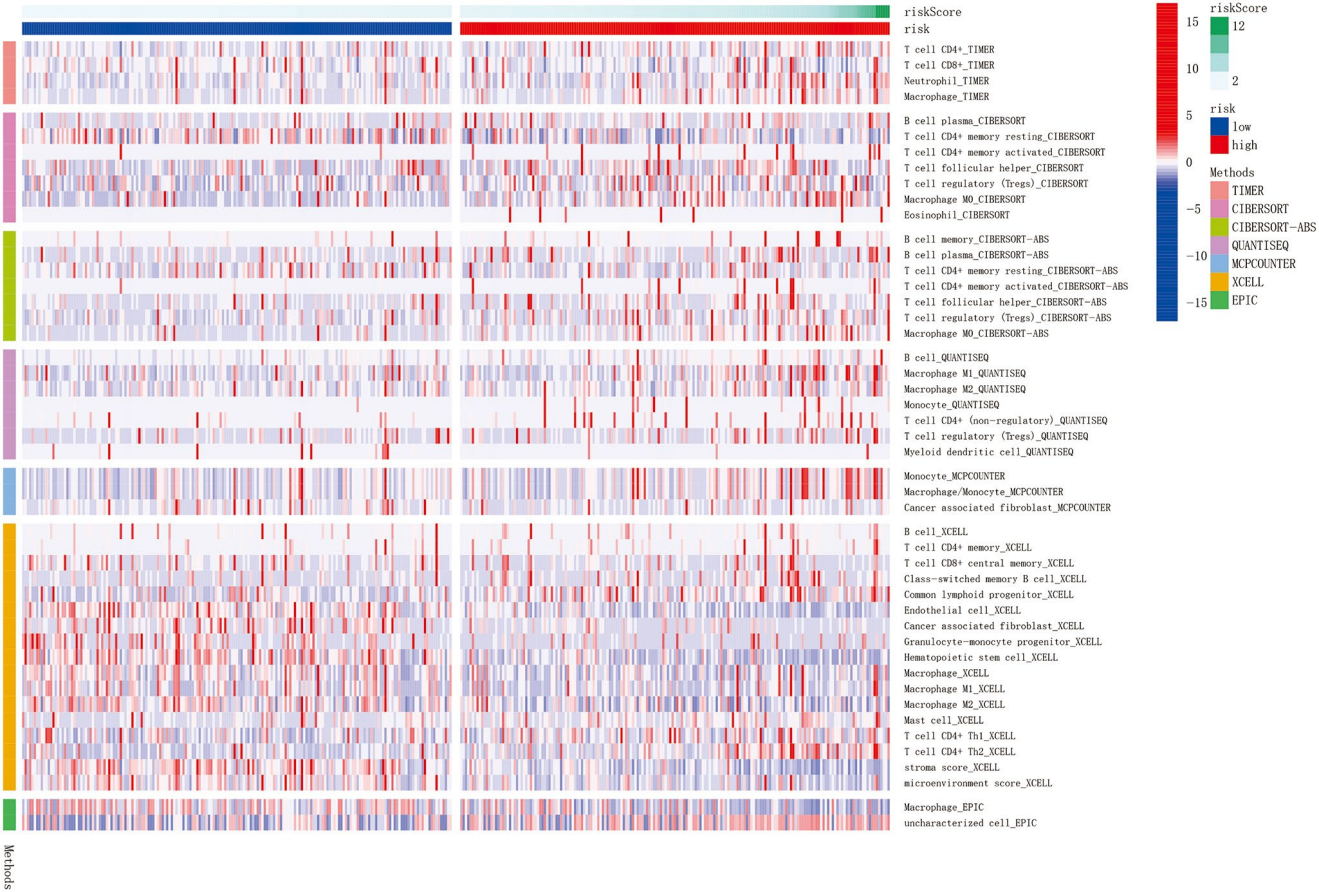
**The immune cell infiltration landscape in HCC.** Heatmap for immune cell infiltration landscape using TIMER, CIBERSORT, CIBERSORTABS, QUANTISEQ, MCPCOUNTER, XCELL, and EPIC algorithms in high and low risk groups (blue: low expression; red: high expression). Only items with significant differences will be presented, *P*-value < 0.05 was controlled.

**Figure 9:**
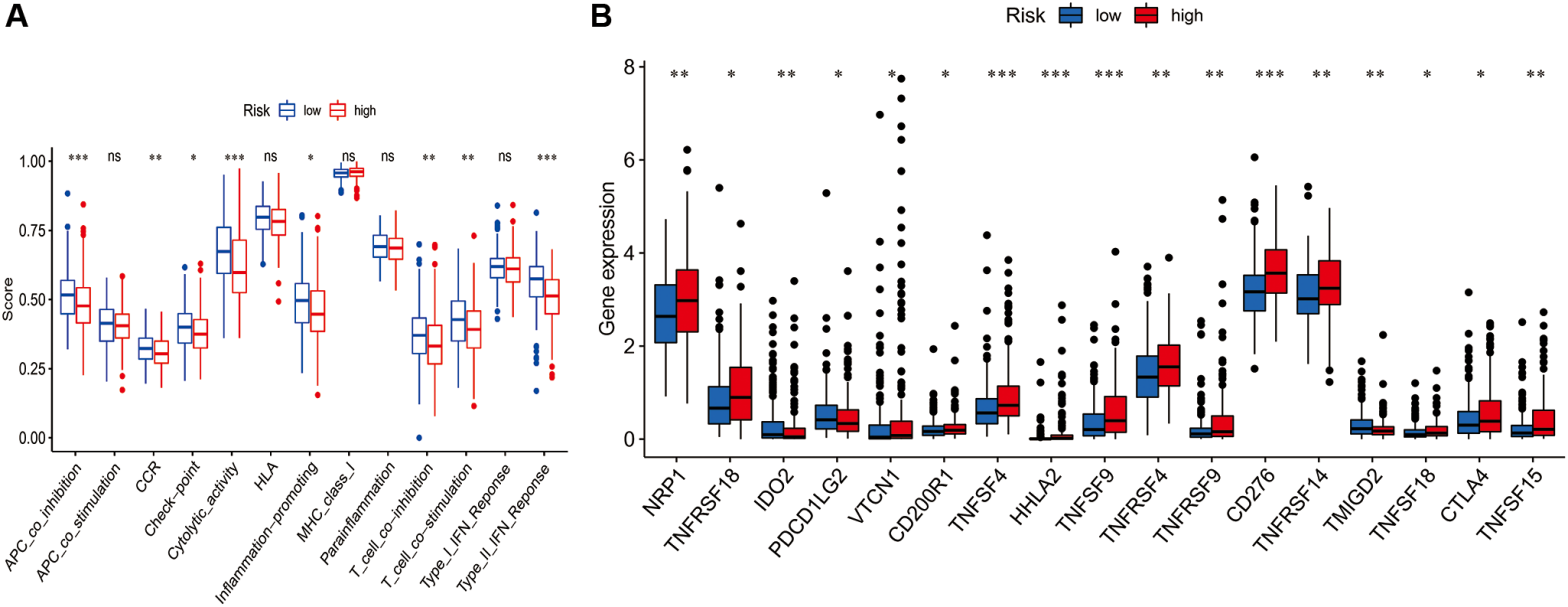
**Comparison of the single sample gene set enrichment analysis (ssGSEA) scores for immune-related functions and immune checkpoints between different risk groups.** (**A**) ssGSEA for the immune functions between high-risk (red box) and low-risk (blue box) groups HCC patients. (**B**) The expression levels of immune checkpoints between high-risk (red box) and low-risk (blue box) groups HCC patients. (^*^P < 0.05, ^**^P < 0.01, and ^***^P < 0.001).

TIMER, CIBERSORT, CIBERSORTABS, QUANTISEQ, MCPCOUNTER, XCELL, and EPIC algorithms were used to examine the immune cell and pathway profiles in the signature-identified high-risk and low-risk groups (Figure 8, all *P* < 0.05). Based on single sample gene set enrichment analysis (ssGSEA) of TCGA-HCC data, correlation analysis between immune cell subgroups and associated functions showed that T cell functions such as APC co-inhibition, CCR, checkpoint, cytolytic activity, inflammation promoting, type II IFN response, T cell co-stimulation, T cell co-inhibition response were markedly different between the high- and low-risk groups, as shown in Figure 9A (*P* < 0.05). In view of the significance of immunotherapies based on checkpoint inhibitors, the distinction in expressing immune checkpoints between the two groups was further explored. The high-risk group expressed more pervasive immune checkpoint molecules such as NRP1, CD276, TNFRSF14, and TNFRSF4 than the low-risk group (Figure 9B, *P* < 0.05).

### Validation of genes expression levels in cell lines

**Figure 10:**
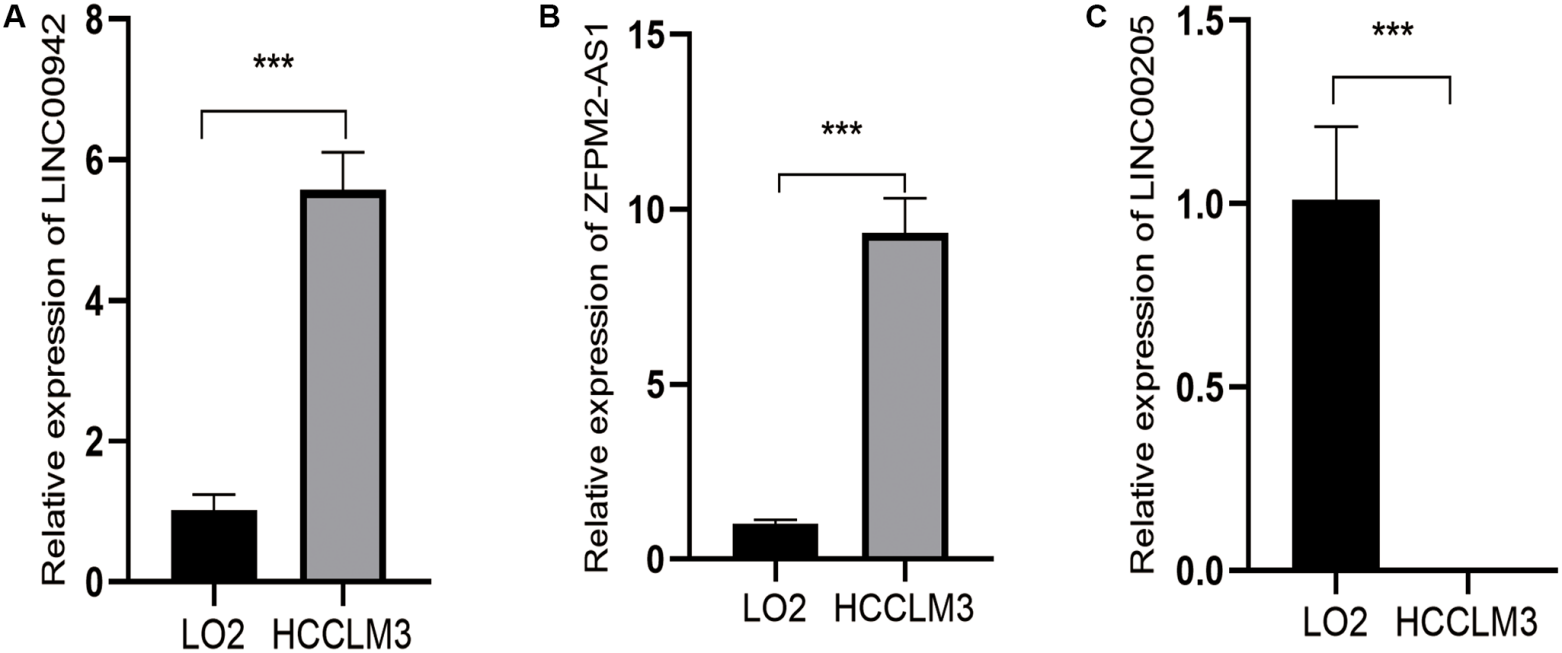
**Expression levels of three ferroptosis-related lncRNAs of prognostic signature in HCCLM3 and LO2 by q-PCR.** (**A**) Relative expression levels of LINC00942 between LO2 and HCCLM3. (**B**) Relative expression levels of ZFPM2-AS1 between LO2 and HCCLM3. (**C**) Relative expression levels of LINC00205 between LO2 and HCCLM3. (^***^P < 0.001).

Compared with normal human hepatic epithelial cell lines LO2, LINC00942 and ZFPM2-AS1 were remarkedly upregulated in HCCLM3 cell lines (Figure 10A, 10B, *P* < 0.001), while LINC00205 was significantly downregulated (Figure 10C, *P* < 0.001).

## DISCUSSION

In this study, we first identified 781 ferroptosis-associated lncRNAs, and then, 66 remarkable ferroptosis-associated lncRNAs were identified. Eventually, a seventeen-ferroptosis-associated lncRNA signature model was established. Following that, based on the medium risk score, HCC patients were separated into low-risk and high-risk groups with significantly different OS. Moreover, the seventeen-ferroptosis-related lncRNA signature model was shown to be an independent prediction factor for HCC after correcting for traditional clinical risk indicators. This result suggested that the seventeen-ferroptosis-related lncRNA signature could reliably predict the prognosis of HCC patients. Next, the functions of immune infiltrating cells in the tumor microenvironment and immune checkpoint inhibitors (ICIs) in HCC prognosis were investigated. In the ferroptosis signaling pathways, our research discovered a possible biomarker and therapeutic target. Finally, we utilized q-PCR to validate the risk model.

HCC is an extremely heterogeneous malignancy, which adds to the difficulty in predicting prognosis [24]. Ferroptosis is becoming more well regarded as a factor in the prognosis of patients with HCC and other malignancies [25–27]. The impact of ferroptosis on tumor development and therapy has been the subject of many studies. At the same time, studies found that lncRNAs play a significant part in the prognosis of HCC, which will be expected to be a possible and valid molecular target for the treatment of HCC [28, 29]. Currently, a host of ferroptosis-based and lncRNA-based prognostic signature models for tumors have been reported [30–32]. Notably, new research shows that several lncRNAs can play an important role in regulating the occurrence and development of diseases through promoting ferroptosis [33–35]. At present, the ferroptosis-associated lncRNA prognostic signature models have been reported in other malignancies [36–38]. However, there was rarely research about the ferroptosis-associated lncRNA prognostic signature model in HCC. In this work, we firstly established a seventeen-ferroptosis-associated lncRNA prognostic signature model, which could reliably predict the prognosis of HCC patients.

In this study, a seventeen-ferroptosis-associated lncRNA signature model was established, including twelve high-risk genes (POLH-AS1, SNHG10, AL139384.1, AL928654.1, AL603839.3, MKLN1-AS, AC090772.3, ZFPM2-AS1, AP001469.3, AC012073.1, AL031985.3, and LINC00942) and five low-risk genes (AC099850.1, LINC00205, AC026401.3, AC145207.8, and SNHG21). Among the high-risk genes, studies have reported that HCC patients with high expressions of SNHG10 [39], MKLN1-AS [40], ZFPM2-AS1 [41] and AL031985.3 [42] were associated with shorter OS and worse prognosis, which are consistent with our findings. Meanwhile, in lung adenocarcinoma, patients with higher LINC00942 demonstrated poor prognosis [43]. Interestingly, study has reported that HCC patients with higher expression of LINC00205 showed worse prognosis, and LINC00205 increases the proliferation, migration, and invasion of HCC cells [44]. However, LINC00205 was a low-risk gene in our study. We think more experimental studies are necessary to explain the incongruous effects of LINC00205 in the future. Other risk genes have not been investigated in tumors, and our findings may help and guide future research.

As we all know, tumor staging and tumor grading are significant elements to be consider when predicting the prognosis of patients with HCC. At present, many staging systems for the prognostic prediction of HCC patients have been devise, such as the American Joint Committee on Cancer (AJCC)-TNM which has limited prognostic prediction value of HCC patients and is commonly utilized by surgeons [45]. Interestingly, we found that clinical features can also predict the OS of HCC patients. However, the predicted effect of the risk model was better than the clinicopathological features by multivariate Cox regression analysis. At the same time, the AUC of risk score was higher than clinical features, indicating that the risk model is better than clinical characteristics in the prediction of prognosis of HCC. Thus, our findings revealed that the novel seventeen-ferroptosis-related lncRNA signature was robustly predictive of OS in HCC patients.

Several abnormal signaling pathways in HCC have been identified in recent research [46]. Some of these abnormal signals may be used to identify novel molecular targets for new therapies, such as Wnt signaling pathway, P53 signaling pathway, and PI3K/AKT-pathway [47]. In this work, we found that immunoregulatory pathways are different in the high-risk subgroup and the low-risk subgroup, which may be used to guide future treatment of HCC. In addition, the anti-tumor immunity of patients in the high-risk and low-risk groups is inconsistent, which may also help guide the treatment of HCC patients in the future.

At present, immunotherapy has emerged as a viable new therapeutic option for HCC [48]. However, the majority of patients did not react to immune checkpoint blockade immunotherapy [49]. The induction of ferroptosis is closely linked to anti-tumor immunity, not only engaging in tumor cell destruction through ICI-activated T cells, but also directly altering the function of diverse immune cells, implying the prospect of cancer synergistic therapy [50]. The combination of ferroptosis and ICIs can improve anti-tumor activity in a synergistical way, even in ICI-resistant types [51]. Currently, studies on the relationship between ICI and ferroptosis remain rare. Hence, a seventeen-ferroptosis-related lncRNA signature was constructed to explore the link between ferroptosis and ICIs. In our study, the expression levels of most ICIs in high-risk subgroup were higher compared with low-risk subgroup. This suggested that the seventeen-ferroptosis-related lncRNA signature might be used to forecast the level of ICIs expression and could be used to guide immune checkpoint blockade immunotherapy. In high-risk HCC patients, combining ICIs with ferroptosis inducers may promote malignant cell ferroptosis, thereby improving overall prognosis. Thus, this combination of ICIs and ferroptosis inducers might lead to novel therapy options for HCC patients in the future.

Our research has several limitations. First, the study data is from the TCGA public database, and our model needs to be checked further with prospective, multi-center, and practical data. Secondly, considering that clinical samples were not used to verify the research results, the reliability of our results is uncertain. In addition, the findings should be utilized carefully given the limitations of clinical data.

## CONCLUSIONS

In summary, our study shows that seventeen-ferroptosis-related lncRNA could precisely predict the prognosis of HCC patients. In addition, this research might provide clues for improving anti-tumor immunity and supplying novel therapy strategies for HCC.

## Supplementary Materials

Supplementary Tables 1, 3-7

Supplementary Table 2
