# Supplementary material for: Ferroptosis-related long non-coding RNA signature predicts the prognosis of hepatocellular carcinoma
Source: Aging (Albany NY). 2022 May 12;14(9):4069–84. doi: 10.18632/aging.204073 (PMC9134948; doi:10.18632/aging.204073)
Supplement: Supplementary Table 2 [file aging-14-204073-s002.pdf]

SUPPLEMENTARY TABLE

Supplementary Table 2. The primer sequences for q-PCR.

Gene	Primer Sequence
LINC00205	F: 5′-AGCCGTTCGTCTTTACCTGG-3′
	R: 5′-TCCAGGAGGACTCATGGGAG-3′
LINC00942	F: 5′-AGGAGCTGGCAAGACCTCTA-3′
	R: 5′-GTTCAGTGACGCAGAATCGC-3′
ZFPM2-AS1	F: 5′-CCCAGGGAGAGTATGGAGTGA-3′
	R: 5′-AGTTGCAAGATGACGCTCAGT-3′
GAPDH	F: 5′-TGACTTCAACAGCGACACCCA-3′
	R: 5′-CACCCTGTTGCTGTAGCCAAA-3′
